# A Telemedicine-Based Registration System for the Management of Renal Anemia in Patients on Maintenance Hemodialysis: Multicenter Study

**DOI:** 10.2196/13168

**Published:** 2019-05-08

**Authors:** Zhaohui Ni, Haijiao Jin, Gengru Jiang, Niansong Wang, Ai Peng, Zhiyong Guo, Shoujun Bai, Rong Zhou, Jianrao Lu, Yi Wang, Ying Li, Shougang Zhuang, Chen Yu, Yueyi Deng, Huimin Jin, Xudong Xu, Junli Zhang, Junli Zhao, Xiuzhi Yu, Xiaoxia Wang, Liming Zhang, Jianying Niu, Kun Liu, Xiaorong Bao, Qin Wang, Jun Ma, Chun Hu, Xiujuan Zang, Qing Yu

**Affiliations:** 1 Department of Nephrology, Renji Hospital School of Medicine, Shanghai Jiao Tong University Shanghai China; 2 Clinical Research Center Shanghai Jiao Tong University School of Medicine Shanghai China; 3 Department of Nephrology Xin Hua Hospital Affiliated to Shanghai Jiao Tong University School of Medicine Shanghai China; 4 Department of Nephrology The Sixth People's Hospital Affiliated to Shanghai Jiao Tong University Shanghai China; 5 Department of Nephrology Tenth People's Hospital of Tongji University Shanghai China; 6 Department of Nephrology Changhai Hospital of Shanghai Shanghai China; 7 Department of Nephrology Qingpu Branch of Zhongshan Hospital Affiliated to Fudan University Shanghai China; 8 Department of Nephrology Yangpu Hospital Affiliated to Tongji University Shanghai China; 9 Department of Nephrology Seventh People's Hospital of Shanghai University of Traditional Chinese Medicine Shanghai China; 10 Department of Nephrology Yueyang Hospital of Integrated Traditional Chinese Medicine and Western Medicine Affiliated to Shanghai University of Traditional Chinese Medicine Shanghai China; 11 Department of Nephrology Central Hospital of Shanghai Jiading District Shanghai China; 12 Department of Nephrology Shanghai East Hospital Shanghai China; 13 Department of Nephrology Tongji Hospital Tongji University School of Medicine Shanghai China; 14 Department of Nephrology Longhua Hospital Shanghai University of Traditional Chinese Medicine Shanghai China; 15 Department of Nephrology Shanghai Pudong Hospital Shanghai China; 16 Department of Nephrology Minhang Hospital Fudan University Shanghai China; 17 Department of Nephrology Jiulingwu Hospital Shanghai China; 18 Department of Nephrology Shanghai Pudong New District Zhoupu Hospital Shanghai China; 19 Department of Nephrology Navy Characteristic Medical Center Shanghai China; 20 Department of Nephrology Tongren Hospital Shanghai Jiao Tong University School of Medicine Shanghai China; 21 Department of Nephrology Zhabei Central Hospital, Jingan District Shanghai China; 22 Department of Nephrology The Fifth People's Hospital of Shanghai Fudan University Shanghai China; 23 Department of Nephrology Jinshan Branch of Shanghai No. 6 People's Hospital Shanghai China; 24 Department of Nephrology Jinshan Hospital Fudan University Shanghai China; 25 Department of Nephrology and Rheumatology Shanghai Fengxian Central Hospital Shanghai China; 26 Department of Nephrology Jingan District Central Hospital of Shanghai Shanghai China; 27 Department of Nephrology No. 9 People Hospital Affiliated to Shanghai Jiao Tong University School of Medicine Shanghai China; 28 Department of Nephrology Shanghai Songjiang District Central Hospital Shanghai China; 29 Department of Nephrology Shanghai General Hospital Shanghai China

**Keywords:** telemedicine, dialysis registration system, hemodialysis, renal anemia, end-stage renal disease

## Abstract

**Background:**

Renal anemia is one of the most important complications in patients on maintenance hemodialysis (MHD). Telehealth-based dialysis registration systems have the advantage of real-time monitoring and have gradually been applied to the management of chronic diseases.

**Objective:**

The objective of our study was to evaluate the impact of a telehealth-based dialysis registration system on patients on MHD in terms of renal anemia control.

**Methods:**

The Red China project aimed to develop a dialysis registration system based on the WeChat mobile platform. Demographic and baseline laboratory parameters such as age, gender, primary disease, dialysis age, and baseline creatinine levels were recorded using this system. In addition, the hemoglobin and hematocrit levels were recorded monthly. The platform then generated a hemoglobin and hematocrit statistics report for each hemodialysis center monthly, including the detection rate, target rate, and distribution of hemoglobin and released it to physicians via the WeChat mobile phone app. The physicians were then able to treat the individual’s anemia appropriately by changing the doses of erythropoiesis-stimulating agents or iron use on the basis of this report. We analyzed the demographic and baseline laboratory parameters, detection rate, target rate, and average level and distribution of hemoglobin 28 months after the launch of the project.

**Results:**

A total of 8392 patients on MHD from 28 hemodialysis centers in Shanghai were enrolled from June 2015 to October 2017. The detection rate of hemoglobin increased from 54.18% to 73.61% (*P*<.001), the target rate of hemoglobin increased from 47.55% to 56.07% (*P*<.001), and the mean level of hemoglobin increased from 10.83 (SD 1. 60) g/dL to 11.07 (SD 1.60) g/dL (*P*<.001). In addition, the proportion of patients with hemoglobin levels ≥11 g/dL but <13 g/dL increased from 40.40% to 47.48%.

**Conclusions:**

This telehealth-based dialysis registration system can provide timely reporting of the anemia status in patients on MHD, which may improve the awareness of anemia and the attention to and compliance with anemia monitoring.

## Introduction

Telehealth technologies offer advantages of accessibility, convenience, and time-effectiveness and are thus being increasingly adopted to aid the management of long-term conditions worldwide. Increasing evidence has demonstrated promising results for telecare or telehealth medicine in the management of diabetes, heart failure, asthma, chronic obstructive pulmonary disease, and cancer [1]; however, data on its use in patients on maintenance hemodialysis (MHD) are still sparse.

Recent Quality and Outcomes Framework Disease Register data comparing data from 2006-2007 to 2010-2011 showed a 45% increase in the prevalence of chronic kidney disease, second only to the increase in cancer (79%) [2]. The management of this long-term condition is increasingly challenging when it develops into end-stage renal disease (ESRD) or requires MHD. Renal anemia is extremely common among patients on MHD and often underlies symptoms including fatigue, depression, reduced exercise tolerance, and dyspnea; increased morbidity and mortality related to cardiovascular disease; an increased risk of hospitalization; and an increased length of hospital stay. Patient mortality and hospitalization risks were shown to decrease by 10%-12% for every 1 g/dL increase in mean facility-level hemoglobin [3]. MHD patients should thus be monitored for anemia in a timely manner and managed carefully; according to the Kidney Disease Improving Global Outcomes (KDIGO) guidelines, hemoglobin levels should be monitored at least monthly [4]. However, the actual management is still not satisfactory. Data from the Dialysis Outcomes and Practice Patterns Study (DOPPS) showed that more frequent hemoglobin monitoring was associated with lower facility-level variations in the hemoglobin levels [5]. Data from Chinese DOPPS facilities also showed that a large proportion of patients on MHD did not meet the expressed hemoglobin target and that less frequent and substantial increases in the doses of erythropoiesis-stimulating agents (ESAs) were associated with hemoglobin levels <9 g/dL [6]. Another cohort study enrolled a total of 2388 patients with ESRD (1775 patients on MHD) from nine centers in the largest dialysis facilities in six cities around China and found that about 60% of the patients did not reach the hemoglobin target of 11 g/dL, even though 85.0% of them were treated with erythropoietin [7].

Several renal dialysis registration data systems exist worldwide, including the United States Renal Data System (USRDS), the European Renal-European Dialysis and Transplantation Association Registry, the Australia and New Zealand Dialysis and Transplant Registry, the State of Chronic Dialysis Therapy in Japan, the Hong Kong Renal registration, and the China National Renal Data System. However, these registration systems collect data from dialysis units and issue a dialysis report every year only for quality measures. In China, a registration system that can reflect the dynamic, real-time anemia status of patients on hemodialysis is still lacking, thus preventing their timely treatment.

We therefore established the Red China project using telehealth technology in June 2015 with the aim of improving renal anemia in patients on MHD, allowing timely reporting to nephrologists, facilitating the early recognition and resolution of anemia, and benefiting the long-term prognosis of patients on MHD.

## Methods

### Participants

The Red China project developed a dialysis registration system based on the WeChat mobile platform. All patients with ESRD undergoing MHD at the 28 centers between June 1, 2015, and October 31, 2017, were enrolled in the study. There were no exclusion criteria, apart from patients not willing to participate. Demographic and baseline laboratory parameters such as age, gender, primary disease, dialysis age, and baseline creatinine levels were recorded in this system. Hemoglobin and hematocrit levels were recorded monthly. We analyzed the demographic and baseline laboratory parameters and the detection rate, target rate, average level, and distribution of hemoglobin from June 2015 to October 2017 after the launch of the project. Detection rate refers to the proportion of patients for whom the hemoglobin level was recorded for one month, and target rate refers to the proportion of patients who achieved a hemoglobin level of 11g/dL in that one month.

### Telehealth System

The project was developed using an online platform based on WeChat, currently regarded as the most popular instant-messaging platform in China. This app is the largest social app in China and has met the requirements of international authoritative certification standards in terms of information security. The platform aimed to help medical staff record and monitor hemoglobin levels, hematocrit levels, and other physiological indicators in real time.

### Clinical User Interface

Data entry was conducted by full-time personnel with research qualifications. The hemoglobin data were collected from laboratories in each hemodialysis center. The platform generated monthly hemoglobin and hematocrit statistics reports for each hemodialysis center, including the detection rate, target rate, and distribution of hemoglobin. The system highlighted patients who were outside the target and released this information to physicians via the WeChat mobile phone app. The physicians were then able to adjust the patient’s treatment to resolve their anemia individually, on the basis of this report.

### Statistical Analysis

Baseline characteristics were expressed as the mean (SD) for normally distributed data and as frequencies and percentages for categorical data. Comparisons between baseline and 28 months after the project were performed using paired *t* tests or Chi-square tests, as applicable. All analyses were performed using SPSS for Windows (version 19.0; SPSS, Inc, Chicago, IL). A *P* value <.05 was considered to be statistically significant.

## Results

### Baseline Characteristics

This study included 8392 patients on MHD from 28 hemodialysis centers in Shanghai. The baseline characteristics are shown in Table 1.

**Table 1:** Baseline characteristics of patients on maintenance hemodialysis.

Characteristic		Value
Sex - men, n (%)		5059 (60.28)
Age (years), mean (SD)		60.5 (13.7)
**Dialysis duration, n (%)**		
	<3 months	1220 (14.54)
	3 months to 1 year	3359 (40.02)
	1 to 5 years	3029 (36.09)
	5 to 10 years	744 (8.87)
	>10 years	40 (0.48)
**Primary disease, n (%)**		
	Glomerulonephritis	3880 (46.23)
	Diabetic nephropathy	781 (9.31)
	Hypertensive nephrosclerosis	843 (10.05)
	Polycystic kidney disease	251 (2.99)
	Others	2002 (23.86)
	Unknown	635 (7.57)
Serum creatinine level (µmol/L), mean (SD)		853.43 (341.59)
Hemoglobin level (g/L), mean (SD)		10.83 (1.60)

### Detection Rate and Target Rate of Hemoglobin

**Figure 1:**
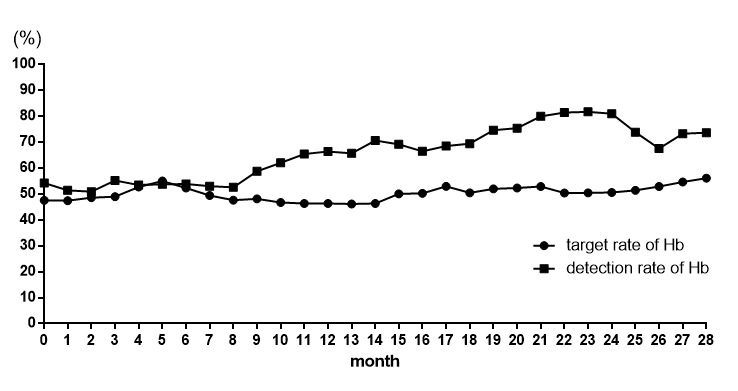
Detection and target rates of hemoglobin in patients on maintenance hemodialysis. Hb: hemoglobin.

The detection and target rates of hemoglobin were both significantly higher in the 28 months after the project was set up compared with the period before the beginning of the project (detection rates: 73.61% vs 54.18%; target rates: 56.07% vs 47.55%, both *P*<.001; Figure 1).

### Improvements in Hemoglobin and Hematocrit Levels

Hemoglobin and hematocrit levels were both significantly higher in the 28 months after the project was set up compared to the period before the start of the project (hemoglobin: mean 11.07, SD 1.60 g/dL vs mean 10.83, SD 1.60 g/dL; hematocrit: mean 34.08%, SD 4.89% vs mean 33.51%, SD 5.12%; both *P*<.001).

### Distribution of Hemoglobin

**Figure 2:**
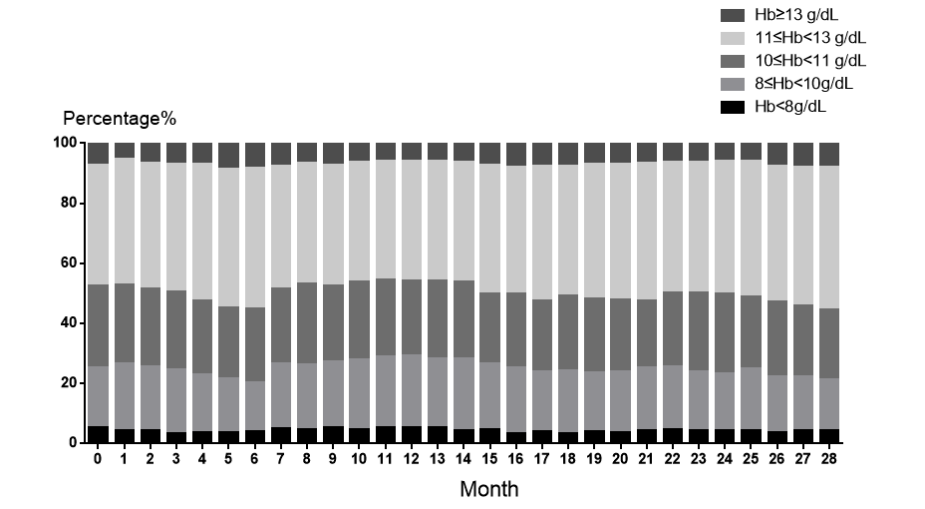
Distribution of hemoglobin in patients on maintenance hemodialysis. Hb: hemoglobin.

The monthly distribution of hemoglobin in patients on MHD is shown in Figure 2. During the 28-month follow-up, the proportion of patients with hemoglobin levels ≥8 g/dL but <10 g/dL decreased from 20.14% to 17.07%, the proportion of patients with hemoglobin levels <8 g/dL decreased from 4.96% to 4.08%, and the proportion of patients with hemoglobin levels ≥11 but <13 g/dL increased from 40.40% to 47.48%.

## Discussion

Recently, there has been mounting evidence of the feasibility of smartphone apps in remote monitoring and flexible follow-up of patients in western countries [8-10]. However, the experience of using a smartphone app in the management of chronic diseases in China is limited. This study is the first in China to use telehealth technology to promote the management of renal anemia, assess user acceptability, and collect data on patients with ESRD on MHD.

The prevalence of ESRD is increasing worldwide. According to Nanjing Urban Employee Basic Medical Insurance data, the prevalence of ESRD is expected to increase by approximately 1.95% annually from 2015 to 2025, with a predictive value of 1505 per million population in 2025 [2], representing high financial and public health burdens. Furthermore, the increasing number of patients with ESRD accessing hemodialysis is associated with increasing challenges in terms of managing the accompanying renal anemia. Anemia is extremely common among dialysis patients and underlies a range of symptoms including fatigue, depression, reduced exercise tolerance, and dyspnea; increased morbidity and mortality related to cardiovascular disease; an increased risk of hospitalization; and a longer hospital stay. According to a 2017 USRDS report, the mean hemoglobin level in ESRD patients was 9.5 g/dL [11], while the KDIGO guidelines, European Best Practices Guideline, and The National Kidney Foundation Kidney Disease Outcome Quality Initiative guidelines recommend a target hemoglobin level of 11-12 g/dL in patients on MHD [4,12,13].

Telehealth technologies offer advantages of accessibility, convenience, and time effectiveness and are thus being increasingly adopted to aid the management of long-term conditions worldwide. Increasing evidence has indicated promising results for telecare and telehealth medicine in the management of diabetes, heart failure, asthma, chronic obstructive pulmonary disease, and cancer [1] and even chronic kidney disease and peritoneal dialysis [14-16]. Sobrinho et al reported that a mobile health app that aimed at assisting in the early diagnosis and self-monitoring of disease progression in patients with chronic kidney disease was associated with quality attributes such as safety, effectiveness, and usability [14]. Telemedicine is also a promising new tool for the remote management of automated peritoneal dialysis, allowing timely intervention prior to the development of more significant problems, reducing the frequency of in-person visits for emergency problems, and reducing health care resource utilization and associated costs [17-19]. However, data on its use in patients on MHD, especially for management of renal anemia, are still sparse.

We therefore developed the Red China project using telehealth technology in June 2015 with the aim of allowing timely reporting to nephrologists, to facilitate the early recognition and resolution of anemia and thus improve the long-term prognosis in patients on dialysis. Similar to the American practice, the Red China project collects and reports the patient hemoglobin test data on a regular basis, prompting the clinician to personally adjust the anemia management medication for patients on MHD, which can reduce the workload of medical staff. In addition to individualized management of patients, the project monitors the compliance status of the patient population in the dialysis center, assists clinicians in overall analysis, and improves MHD patients’ hemoglobin level.

Our results showed that achieved rate of target hemoglobin levels in patients on MHD increased significantly from 47.55% to 56.07% during the 28 months following the introduction of the project, compared with another cohort study in China that showed a target rate of 40% [7]. Our results also showed that the proportion of patients with a hemoglobin level <8 g/dL decreased from 4.96% to 4.08% following the start of the project, compared with 12% in China’s DOPPS research [6], 31.7% in Japan’s DOPPS study [20], and 8.7% in North America’s DOPPS study [20,21]. The mean hemoglobin level in this study increased from 10.83 (SD 1.60) g/dL to 11.07 (SD 1.60) g/dL, compared with the mean hemoglobin levels of 10.5 (SD 2.0) g/dL, 10.4 (SD 1.2) g/dL, and 11.5 (SD 1.2) g/dL in China, Japan, and North America, respectively. Although the Red China project has the potential to help clinicians improve anemia in patients on hemodialysis, there is a gap in performance between Shanghai and developed countries such as Japan and North America. Notably, we did not observe a consistent improvement in the hemoglobin levels over baseline for several months. This could be because many other factors played an important role in modulation of the hemoglobin levels, including dialysis adequacy, nutrient status, infection, and chronic gastrointestinal bleeding, which were not recorded in this project. In addition, management of renal anemia via the app took time to yield benefits.

In terms of privacy and security, the Red China project follows the “National Standard of the People's Republic of China: GB/T 35273-2017 Personal Information Security Specification of Information Security Technology.” When collecting information, full informed consent was obtained from all patients on MHD and data entry was conducted by full-time personnel with research qualifications. Only researchers have access to the patient data in the center. The data are deidentified, and security measures such as encryption are used for data transmission and storage. The Red China project uses the largest social app in China—WeChat—for patient management. The WeChat platform uses information security technologies such as encryption and anonymization to ensure information security. WeChat has passed the assessment and filing of national network security–level protection and has met the requirements of international authoritative certification standards. The Red China project uses the Alibaba Cloud server to transmit normal and secure traffic back to the server by conducting recognition of malicious features and protecting the service traffic of the app; through the website HTTPS encryption, it can prevent hijacking and tampering, avoid malicious invasion of the website server, and ensure the security of core data.

This study had several limitations. We did not include information on serum iron levels, total iron-binding capacity, transferrin saturation, ferritin levels, or use of drugs such as iron and ESA. However, we aim to improve this platform and include other laboratory parameters such as iron metabolism indicators as well as iron and ESA use in the future. In addition, this telemedicine system has been limited to the physicians, and we believe it would be useful to send the information to patients as well and provide patient education to improve clinical outcomes in the future.

Telehealth technology offers a tool for improved monitoring and calibration of anemia to meet the recommended targets. This telemedicine system could be also used to analyze the reason why some patients could not meet the target and to help develop strategies to improve patient outcomes for anemia management as well as other clinical parameters such as dietary intervention for control of phosphate levels and blood pressure. Further studies in emerging dialysis practices serving large numbers of patients are needed to determine the effects of this technology on improving the achievement of anemia targets as well as the associations with patient outcomes.

In conclusion, telehealth technology offers a promising, feasible, and accessible tool for improving the management of renal anemia in patients on MHD.

## Abbreviations

DOPPS: Dialysis Outcomes and Practice Patterns Study
ESA: erythropoiesis-stimulating agents
ESRD: end-stage renal disease
KDIGO: Kidney Disease Improving Global Outcomes
MHD: maintenance hemodialysis
USRDS: United States Renal Data System
